# Psoas Muscle Volume as an Opportunistic Diagnostic Tool to Assess Sarcopenia in Patients with Hip Fractures: A Retrospective Cohort Study

**DOI:** 10.3390/jpm11121338

**Published:** 2021-12-09

**Authors:** Sang-Pil So, Bum-Sik Lee, Ji-Wan Kim

**Affiliations:** Department of Orthopaedic Surgery, Asan Medical Center, University of Ulsan College of Medicine, Seoul 05505, Korea; askwhy24@naver.com (S.-P.S.); benton2@naver.com (B.-S.L.)

**Keywords:** hip fracture, psoas muscle, sarcopenia, appendicular skeletal muscle mass

## Abstract

Purpose: This study aims to determine whether the psoas volume measured from a pelvic computed tomography (CT) could be a potential opportunistic diagnostic tool to measure muscle mass and sarcopenia in patients with hip fractures. Methods: This was a retrospective cohort study. In total; 57 consecutive patients diagnosed with hip fractures who underwent surgery were enrolled. A cross-sectional area of the psoas muscle was measured at the lumbar (L) 3 and L4 vertebrae from a pelvic CT for the diagnosis of hip fractures. The psoas muscle volume was calculated with a three-dimensional modeling software program. The appendicular skeletal muscle mass (ASM) and preoperative handgrip strength (HS) were measured. The correlations between the psoas muscle volume/area and ASM/HS were assessed. Data on patient demographics; postoperative complication; length of hospital stay; and Koval scores were also recorded and analyzed with respect to the psoas muscle area/volume. Results: The psoas muscle volume and adjusted values were significantly correlated with ASM; which showed a stronger correlation than the psoas muscle area did at the L3 or L4 level. HS was correlated with the psoas volume or adjusted values; but not with the cross-sectional area of the psoas muscle. Among the adjusted values; the psoas muscle volume adjusted for the patient’s height (m^2^) showed a strongest correlation with ASM and HS. The psoas muscle volume was not significantly correlated with postoperative complications or short-term functional outcomes. Conclusions: The psoas muscle volume measured from a pelvic CT for the diagnosis of hip fractures showed a stronger correlation with ASM and HS than the cross-sectional area did. Therefore; the psoas muscle volume could be a potential diagnostic tool to assess the quantity of the skeletal muscle in patients with hip fractures without an additional examination.

## 1. Introduction

Sarcopenia is a progressive and generalized skeletal muscle disorder associated with an increased likelihood of adverse outcomes, such as falls, fractures, physical disability, and mortality [1]. In patients with hip fractures, sarcopenia is a significant independent predictor of postoperative clinical outcomes, including mortality, active daily life, mobility, and quality of life [2,3,4,5,6,7,8]. Therefore, more prompt treatments and active rehabilitation options are needed in patients with hip fractures also diagnosed with sarcopenia.

According to the Asian Working Group for Sarcopenia (AWGS), sarcopenia is diagnosed when a patient exhibits a combination of a low muscle quantity and either low muscle strength or physical performance, while severe sarcopenia is diagnosed if the patient exhibits a low muscle quantity, low muscle strength, and low physical performance [9]. According to the AWGS diagnostic criteria, muscle quantity is assessed using the appendicular skeletal muscle mass (ASM), which can be measured through dual-energy X-ray absorptiometry (DXA) or a bioelectrical impedance analysis (BIA). Muscle strength is measured by assessing handgrip strength. However, in patients with acute hip fractures, BIA is challenging to perform due to pain and immobility, and an accurate measurement of muscle quantity and quality is not easily achievable.

The cross-sectional area of the lumbar muscle assessed by computed tomography (CT) or magnetic resonance imaging (MRI) has been actively investigated as a tool to measure muscle mass [10,11]. It can provide a quantification of muscle mass and assess muscle quality, including fatty muscle degeneration. Muscle mass and/or quality can be prognostic factors in various diseases [12,13,14,15]. In hip fractures, three-dimensional (3D) pelvic CT scans have been commonly performed to assess the fracture pattern and plan surgical treatment, and the psoas muscle volume or cross-sectional areas can be measured using a pelvic CT.

In this study, we try to propose for the psoas muscle volume to be assessed by a pelvic CT as a potential opportunistic diagnostic tool without any additional test to assess sarcopenia in patients with immobile hip fractures. We hypothesize that the psoas muscle volume can reflect skeletal muscle quantity better than the cross-sectional area of the psoas muscle, because the psoas muscle is a 3D structure, and the cross-sectional area lacks the standard axial level for measurement. This study analyzes the correlation between the psoas muscle volume/cross-sectional area measured by a 3D pelvic CT and ASM/handgrip strength in patients with hip fractures.

## 2. Materials and Methods

### 2.1. Study Design and Patient Selection

**Figure 1:**
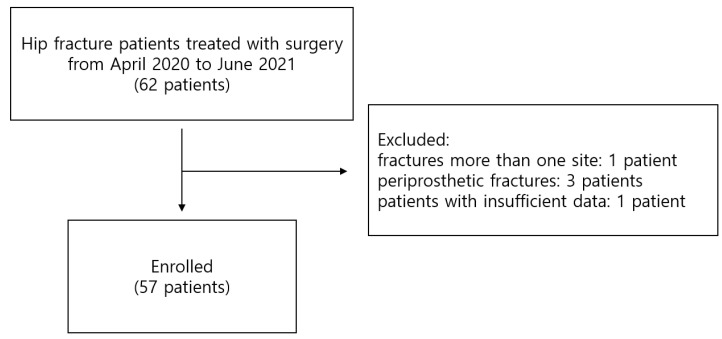
Flowchart of patients enrolled in this study.

This was a single-center retrospective cohort study approved by the institutional review board of the Asan Medical Center (approval no.: 2021–0798; date of approval: 20 May 2021). The inclusion criteria were as follows: (1) patients diagnosed with hip fractures, defined as femoral neck, intertrochanteric, or subtrochanteric fracture and surgically treated from April 2020 to June 2021; (2) low-energy trauma; (3) women aged 55 years or older; (4) 3D pelvic CT scan and body composition analysis (BCA) using DXA performed during the patient’s hospital stay. Patients underwent one of the following surgeries: total hip replacement arthroplasty, bipolar hemiarthroplasty, intramedullary nailing for hip fracture, and multiple screw fixation for femoral neck fracture. We defined low-energy trauma as a fall from a standing height or a height < 1 m [16]. Patients with fractures at more than one site, periprosthetic fracture, or metastatic pathologic fracture were excluded. Patients with insufficient data were also excluded. A total of 62 patients was initially enrolled; 1 patient with fractures at more than one site, 3 patients with periprosthetic fractures, and 1 patient with insufficient data were excluded. A final total of 57 patients was included in the study (Figure 1).

### 2.2. Data Collection

#### 2.2.1. Psoas Muscle Segmentation and Volume Measurement

**Figure 2:**
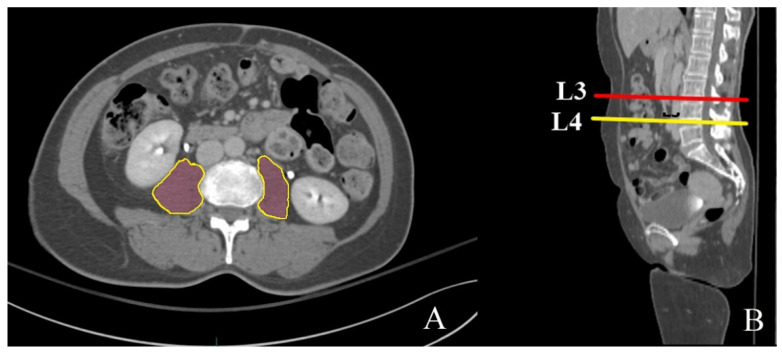
(**A**) Demarcation of the psoas muscle cross-sectional area. (**B**) Measurement level of the psoas muscle at the inferior endplate of the third and fourth lumbar vertebra.

A 3D pelvic CT scan (SOMATOM Definition AS; Siemens, Munich, Germany) was performed with 3 mm thick slices at the emergency room to evaluate the fracture pattern and establish a surgical plan in patients with hip fractures. CT images in the digital imaging and communications in medicine (DICOM) format were imported into a 3D modeling software program (AVIEW Modeler; Coreline Soft, Co., Ltd., Seoul, Korea) to produce 3D samplings of anatomical elements of the psoas muscle. One expert imaging analyzer and one orthopedic surgeon manually demarcated the margin of the psoas muscle together at the level of the inferior endplate of the lumbar (L)3 and the inferior endplate of the L4 vertebrae. The AVIEW Modeler then automatically calculated the cross-sectional area of the psoas muscle at each level (Figure 2). Each patient’s cross-sectional area was adjusted for the patient’s height in m^2^ (PA-L3, PA-L4), which is commonly used to adjust the cross-sectional area of the psoas muscle.

**Figure 3:**
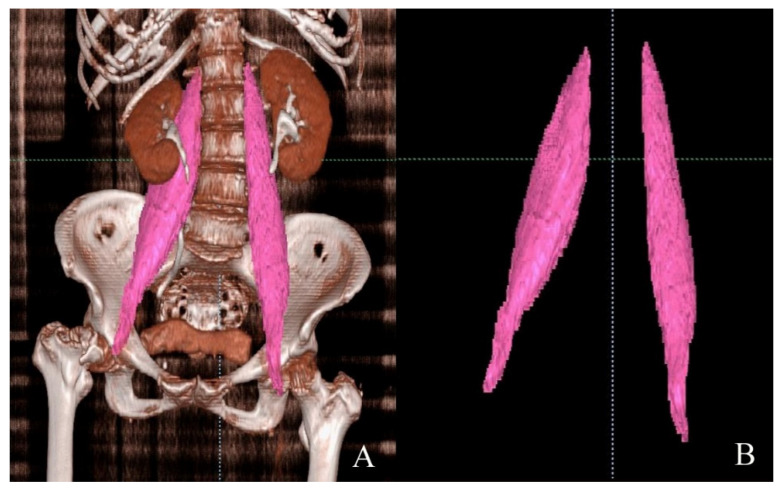
Segmentation of the psoas muscle and measurement of the psoas muscle volume. (**A**) Segmented psoas muscle after manual marking at the margin of the psoas muscle at all axial cuts from the twelfth thoracic vertebra to the lesser trochanter. (**B**) Isolation of psoas muscle from adjacent tissues.

For the segmentation of the psoas muscle, manual markings at the margin of the psoas muscle at all axial images from the level of the twelfth thoracic vertebra to the lesser trochanter were created, and psoas muscle volume was automatically calculated with the AVIEW Modeler (Figure 3). Because there is no established method to date to adjust psoas muscle volume, we used the unadjusted psoas muscle volume (total psoas volume, TPV; TPV1) and tried to adjust the psoas muscle volume for the patient’s height in m (TPV2) as a fundamental unit. Considering that ASM and the cross-sectional area were adjusted for the patient’s height in m^2^, we adjusted the psoas muscle volume for the patient’s height in m^2^ (TPV3). We also adjusted it for the patient’s height in m^3^ (TPV4) because the psoas muscle volume was the measured value of the 3D structure. We used unadjusted (TPV1) and adjusted psoas muscle volumes (TPV2, TPV3, and TPV4) for the analysis.

#### 2.2.2. Measurement of Skeletal Muscle Mass and Handgrip Strength

As a standard method to measure skeletal muscle mass, BCA was performed using DXA (GE Lunar Prodigy; Siemens, Munich, Germany) during the patient’s hospital stay. BCA assessed the mass of fat, muscle, and bone mineral components of each limb and the trunk. ASM was measured as the sum of skeletal muscle mass at both upper and lower extremities, and it was adjusted for the patient’s height in m^2^. According to the AWGS, the diagnostic criteria for low skeletal muscle mass by ASM is < 5.4 kg/m^2^ in women. To assess muscle strength, handgrip strength was measured preoperatively in the supine position with shoulder adduction and 90° of elbow flexion. It was checked at both hands three times per hand with a hand dynamometer (TKK5401; Takei Corporation, Niigata, Japan). The mean value of the three-time measurement was calculated, and the larger value between the left and right hand was used for the analysis. According to the AWGS criteria, low muscle strength was defined as handgrip strength < 18 kg in women.

### 2.3. Functional Outcomes

We collected demographic data, including height, weight, sex, and age. To assess postoperative short-term outcomes, we used postoperative complications and the length of hospital stay. Postoperative complications were classified according to the Clavien–Dindo classification system (Minor: 1–2; Major: 3–5): any deviation from the normal postoperative course 1; normal course altered 2; complications that require intervention of various degrees 3; complications threatening life of patients 4; The death of a patient 5 [17]. To assess the preoperative physical status, we reviewed the American Society of Anesthesiologists (ASA) grade of physical status, which represents the overall health status and is classified into five grades. To assess the walking ability, the preoperative Koval score was used. This describes the walking ability of patients, from 1 (independent community ambulatory) to 7 (nonfunctional ambulator) [18]. Variables related to functional outcomes were analyzed with psoas muscle volume and with ASM and the psoas muscle cross-sectional area, which were previously established methods to measure skeletal muscle mass.

### 2.4. Statistical Analysis

We calculated the mean and 95% confidence interval (CI) for the continuous variables and reported counts and proportions for the ordinal and nominal variables. We used the Pearson correlation test to assess the correlation between continuous variables. Through the Pearson correlation test, the correlation between psoas muscle volume, cross-sectional area, and ASM/handgrip strength was analyzed; the correlation between psoas muscle volume, cross-sectional area, ASM, and length of hospital stay was also assessed. We used the Spearman correlation test to check the correlation between continuous and ordinal variables, such as the correlation between psoas muscle volume, cross-sectional area, ASM, and postoperative complications/ASA grade of physical status/Koval score. Statistical significance was set at *p* < 0.05. Data analysis was carried out using IBM SPSS Statistics for Windows, version 21 (IBM Corp., Armonk, NY, USA).

## 3. Results

### 3.1. Demographic Variables

Detailed patient demographic data are shown in Table 1. The patients’ mean age was 79.5 years (range, 59–93), and the mean value of ASM was 5.4 kg/m^2^ (95% CI, 5.1–5.7). The incidence of low skeletal muscle mass by ASM was 49.1% (28 patients). Preoperative handgrip strength was assessed in 46 patients, with a mean value of 11.7 kg (95% CI, 10.2–13.3), and the rate of a low handgrip strength was 91.3%.

The hip fracture group showed higher preoperative and lower postoperative VAS scores than the control group (*p* = 0.049 and *p* < 0.001, respectively).

### 3.2. Comparisons of ASM between Psoas Muscle Area and Psoas Muscle Volume

ASM was significantly correlated with both the psoas muscle volume and the cross-sectional area. TPV3 showed the highest correlation coefficient, followed by TPV2 and TPV4, while PA-L4 showed the lowest correlation coefficient (Table 2).

### 3.3. Comparisons of HGS between Psoas Muscle Area and Psoas Muscle Volume

Handgrip strength was significantly correlated with the psoas muscle volume, its adjusted values, and ASM. The psoas muscle volume and its adjusted values yielded a higher correlation coefficient with handgrip strength than with ASM. Of these variables, TPV3 showed the strongest Pearson correlation coefficient. However, the psoas muscle cross-sectional areas were not significantly correlated with handgrip strength (Table 3).

### 3.4. Functional Outcomes

According to the Clavien–Dindo classification of postoperative complications, 10 patients did not have complications during hospitalization, while 43 patients had minor complications (Grade 1: 13 patients, Grade 2: 30 patients), and 4 patients had major complications (Grade 3: 4 patients, Grades 4–5: none). Most of the patients with grade two complications developed it due to a postoperative red blood cell transfusion. The mean length of stay was 5.5 d (95% CI, 4.7–6.2). The psoas muscle volume, the cross-sectional area, and ASM were not significantly correlated with postoperative complications, length of hospital stay, ASA grade of physical status, and Koval scores (Table 4).

## 4. Discussion

This study demonstrated that the psoas muscle volume, its adjusted values, and the cross-sectional area at the L3 and L4 level were significantly correlated with ASM and that an adjusted psoas muscle volume showed a higher correlation coefficient than the unadjusted psoas muscle volume and cross-sectional area did. In addition, the psoas muscle volume was significantly correlated with handgrip strength and showed a stronger correlation than with ASM, while the cross-sectional area was not significantly correlated with handgrip strength. These findings suggest that the psoas muscle volume measured by a 3D pelvic CT could be used as a potential opportunistic screening tool for the diagnosis of sarcopenia.

ASM calculated via DXA or BIA has been used as a standard method to measure the quantity of skeletal muscle. However, DXA and BIA have inconsistent results for different instrument brands, and BIA can be influenced by the hydration status of the patient [19,20,21,22,23]. Above all, additional examinations are needed to measure muscle mass in conjunction with either of these methods. In contrast, we could measure the psoas muscle volume and cross-sectional area by pelvic CT without additional examinations in patients with hip fractures.

In the current study, the psoas muscle volume showed a higher correlation with muscle mass measured by ASM or handgrip strength than the cross-sectional area did. Amini et al. suggested that the psoas muscle volume may be better in defining sarcopenia than a single axial image can. They also revealed its association with both short-term and long-term outcomes following the resection of pancreatic cancer [24]. Because the psoas muscle is a 3D structure and the cross-sectional area lacks the standard axial level for measurement, we hypothesized that the psoas muscle volume would have a stronger correlation with ASM than the cross-sectional area does. In addition, we assumed that an adjusted psoas muscle volume would have a stronger correlation with ASM than the unadjusted one would, because we used the adjusted value of ASM. Our results were consistent with our assumption. The psoas muscle volume showed a stronger correlation with ASM than the cross-sectional area did, and, out of these, the adjusted psoas muscle volumes (TPV2, TPV3, and TPV4) showed a higher correlation coefficient than the unadjusted volume did (TPV1). Overall, the results suggest that we could consider using an adjusted psoas muscle volume rather than the cross-sectional area to measure skeletal muscle quantity in patients with a hip fracture without needing additional examinations.

Handgrip strength has been widely used to assess muscle strength [25,26]. Pawel et al. reported an association between the psoas muscle cross-sectional area and handgrip strength [27]. In the current study, the unadjusted total psoas muscle volume and adjusted psoas muscle volumes showed a significant correlation with handgrip strength, while the cross-sectional area was not significantly correlated. In addition, the adjusted psoas muscle volumes (TPV2, TPV3, and TPV4) yielded higher correlation coefficients than the unadjusted volume (TPV1) and ASM did. This implies that we could consider using adjusted psoas muscle volumes (TPV2, TPV3, and TPV4) to reflect muscle strength in patients with a hip fracture without needing additional examinations. Considering the degree of correlation with both ASM and handgrip strength, we propose the psoas muscle volume adjusted by m^2^ (TPV3) as the most reliable parameter to assess skeletal muscle mass and muscle strength simultaneously. However, further studies with larger populations are needed to determine which volume yields the best adjusted value.

The impact of sarcopenia, a well-known predictor of functional outcomes in patients with hip fractures, on clinical was investigated. In these patients, Landi et al. showed an association between sarcopenia and active daily life after discharge from a rehabilitation hospital and after 3 mo., and Byun et al. reported an association between sarcopenia in women and 1 y mortality [2,6]. In the current study, psoas muscle volume and adjusted values did not show a significant correlation with short-term postoperative outcomes, including the grade of postoperative complications and length of hospital stay after surgery. We assumed that as the length of stay after surgery is usually determined by hospital policy, minor complications between patients might not have provoked a significant statistical difference. Regarding the preoperative physical status, there is a debate on the association between sarcopenia defined by the cross-sectional area of the psoas muscle or lumbar muscle and the ASA grade of the physical status [28,29]. There have also been various reports on the association between sarcopenia assessed by ASM and preoperative Koval scores [30,31]. In this study, the psoas muscle volume was not significantly correlated with the preoperative ASA grades and Koval scores. We could consider that the preoperative physical status is affected by various factors, and psoas muscle volume itself might not be enough to show an association with the preoperative general physical status. Further studies with larger sample sizes and long-term follow-up periods are needed.

This study had some limitations. First, it was a retrospective study. When reviewing complications after surgery, a precise comprehension of the situation was difficult. There were also some patients whose data on handgrip strength were unavailable. Second, the sample size was too relatively small to establish the reference value of a low muscle mass and low muscle strength with the psoas muscle volume. Further studies with larger populations are needed to define the criteria of sarcopenia for both men and women. Nonetheless, this study had several strengths. To the best of our knowledge, this study was the first to propose the psoas muscle volume as a tool to assess the strength and quantity of muscle simultaneously without additional examinations in patients with hip fractures, implying that this could be easily implemented in clinical settings. In addition, the values of variables related to the patients were reliable because a single surgeon was more likely to have a consistent surgical technique and postoperative care compared to multiple surgeons. Furthermore, this study dealt with various aspects of patient data, ranging from the preoperative patient status to postoperative complications and length of stay after surgery. Moreover, if the segmentation and measurement of the psoas muscle volume were automated and softwarized by machine learning in the future, a computer program could be used for detecting sarcopenia without needing a person performing the calculations.

## 5. Conclusions

The psoas muscle volume using a pelvic CT of patients with hip fractures showed a stronger correlation with ASM and handgrip strength than the psoas cross-sectional area did. Therefore, the measurement of the psoas muscle volume could be a potential tool to assess the quantity and strength of the skeletal muscle simultaneously in patients with hip fractures without additional examinations.

## Figures and Tables

**Table 1:** Patient demographics.

Characteristic	Value (*n* = 57)
Mean age (y)	79.5 (range, 59–93)
BMI (kg/m^2^)	21.8 (95% CI, 20.8–22.7)
ASM (kg/m^2^)	5.4 (95% CI, 5.1–5.7)
TPV1 (cc)	173.2 (95% CI, 159.9–186.4)
TPV2 (cc/m)	112.3 (95% CI, 104.2–120.4)
TPV3 (cc/m^2^)	72.9 (95% CI, 67.8–78.0)
TPV4 (cc/m^3^)	47.4 (95% CI, 44.1–50.8)
PA-L3 (mm^2^/m^2^)	439.8 (95% CI, 402.2–477.3)
PA-L4 (mm^2^/m^2^)	550.6 (95% CI, 514.1–587.1)

BMI, body mass index; ASM, appendicular skeletal muscle mass; TPV, total psoas volume.

**Table 2:** Pearson correlation test between psoas muscle volume, cross-sectional area, and ASM.

	Volume				Cross-Sectional Area	
ASM (*n* = 57)	TPV1	TPV2	TPV3	TPV4	PA–L3	PA–L4
Correlation coefficient	0.375	0.392	0.396	0.385	0.326	0.292
*p*-value	<0.01	<0.01	<0.01	<0.01	0.01	0.03

ASM, appendicular skeletal muscle mass; TPV, total psoas volume.

**Table 3:** Pearson correlation test between psoas muscle volume, cross-sectional area, ASM, and handgrip strength.

		Volume				Cross-Sectional Area	
Handgrip Strength (*n* = 46)	ASM	TPV1	TPV2	TPV3	TPV4	PA–L3	PA–L4
Correlation coefficient	0.316	0.320	0.333	0.336	0.327	0.071	0.228
*p*-value	0.03	0.03	0.02	0.02	0.03	0.64	0.13

ASM, appendicular skeletal muscle mass; TPV, total psoas volume.

**Table 4:** Spearman correlation test between psoas muscle volume, cross-sectional area, ASM, and grade of complication/ASA grade of physical status/Koval score. Pearson correlation test between psoas muscle volume, cross-sectional area, ASM, and length of hospital stay.

	Grade of Complication (*n* = 57)		Length of Stay (*n* = 57)		ASA Grade (*n* = 57)		Koval Score (*n* = 57)	
	Correlation Coefficient	*p*-Value	Correlation Coefficient	*p*-Value	Correlation Coefficient	*p*-Value	Correlation Coefficient	*p*-Value
ASM	−0.028	0.84	−0.049	0.74	0.057	0.67	−0.028	0.84
TPV1	−0.125	0.36	−0.057	0.70	−0.107	0.43	−0.041	0.76
TPV2	−0.090	0.51	−0.058	0.70	−0.099	0.46	−0.033	0.81
TPV3	−0.047	0.73	−0.056	0.71	−0.062	0.65	−0.013	0.92
TPV4	−0.029	0.83	−0.052	0.73	−0.043	0.75	0.011	0.94
PA-L3	−0.043	0.75	0.034	0.82	0.032	0.81	−0.091	0.50
PA-L4	−0.041	0.76	0.070	0.64	0.136	0.31	−0.095	0.48

ASM, appendicular skeletal muscle mass; ASA, American Society of Anesthesiologists; TPV, total psoas volume.

## Data Availability

The data presented in this study are available upon request from the corresponding author. The data are not publicly available because of the conditions of the ethics committee of our university.
